# The Structure of a CXCR4:Chemokine Complex

**DOI:** 10.3389/fimmu.2015.00282

**Published:** 2015-06-05

**Authors:** Tracy Marie Handel

**Affiliations:** ^1^University of California, San Diego, CA, USA

**Keywords:** CXCR4, metastasis, chemokines, membrane protein structure, protein–protein complex

CXCR4 was the first chemokine receptor to be identified as an HIV coreceptor in 1996 (1). Along with the importance of CXCR4 in development, it was also discovered as a key chemokine receptor in the metastasis of breast (2) and numerous other cancers (3). These were the main reasons that motivated us to pursue structural studies of CXCR4 with synthetic inhibitors and chemokines. However, my laboratory took a rather circuitous route to this goal, and I did a lot of reinventing myself as a scientist along the way.

As background, I got my Ph.D. in chemistry/membrane biophysics at the California Institute of Technology, and then in 1989 accepted a postdoctoral position to do “protein design” with Bill DeGrado at E. I. Du Pont de Nemours (we called it Du Pont University back then, given the amazing freedom we had to do truly basic research). Du Pont (primarily a chemical company) formed a Joint Venture and became Du Pont Merck Pharmaceuticals; and when I was transitioning to a full time employee in 1992, we were tasked with coming up with new therapeutic targets for the expanded pharmaceutical side of the business. I was hired as part of the macromolecular NMR group headed by Peter Domaille, and thus my target choices were biased by some of the exciting work emerging from the structural biology community. This included the first structure of a chemokine, interleukin-8 (IL-8, now CXCL8), which was published in 1990 by Angela Gronenborn’s NMR group at NIH (4). I remember being intrigued by the dimeric structure and thinking (as they described in their paper) that the dimeric architecture of two alpha helices on top of a beta sheet platform might provide a perfect binding site for the IL-8 receptor, as it was reminiscent of the human class I histocompatibility antigen HLA-A2 binding pocket for antigenic peptides. In 1989, two separate groups had cloned the gene for the related CC chemokine, monocyte chemotactic protein-1 (MCP-1, also called MCAF, now CCL2) (5, 6), and although the MCP-1 receptor (CCR2) had not yet been cloned, it looked like this system might be a good target for inflammation. It was consequently adopted as a focus of the Du Pont Merck inflammatory disease group, with the goal of inhibiting the receptor. Inspired by the IL-8 structure and the expectation that MCP-1 would also be a tractable target for NMR, Peter Domaille and I began working on its structure around 1992. We were hoping to obtain the structure of the first CC chemokine, but not surprisingly, the powerhouse NIH group beat us by a long shot and solved MIP-1β in 1994 (7), as did Nick Skelton and Tom Schall at Genentech, who solved the structure of RANTES in 1995 (8). Nevertheless, we persisted, and although I left Du Pont Merck for a faculty position at the University of California Berkeley in 1994, we published the structure of MCP-1 in 1996 (9).

At Berkeley, I continued working on MCP-1 in collaboration with a group at Roche led by Kurt Jarnagin. A major question that arose from the prevalence of dimeric chemokine structures that had been solved was whether they bound receptors as dimers (the prevailing hypothesis) or as monomers. By identifying a mutant that was incapable of dimerizing but was as potent as WT MCP-1 in migration and receptor binding assays, we demonstrated that it bound CCR2 as a monomer (10). This conclusion was consistent with a prior study by Ian Clark-Lewis who had shown that IL-8 was also a functional monomer (11). We also did a fairly comprehensive mutagenesis study of the residues involved in binding and signaling and came up with a model, which was published in 1999 (Figure 1A) (12). Although we never properly docked MCP-1 to the rhodopsin-based model of the receptor, we were qualitatively on the right track of what the structure might look like. However, it was just a model based on mutagenesis data, and I really wanted to determine high-resolution structures of intact receptors with chemokines and/or small molecule antagonists.

**Figure 1:**
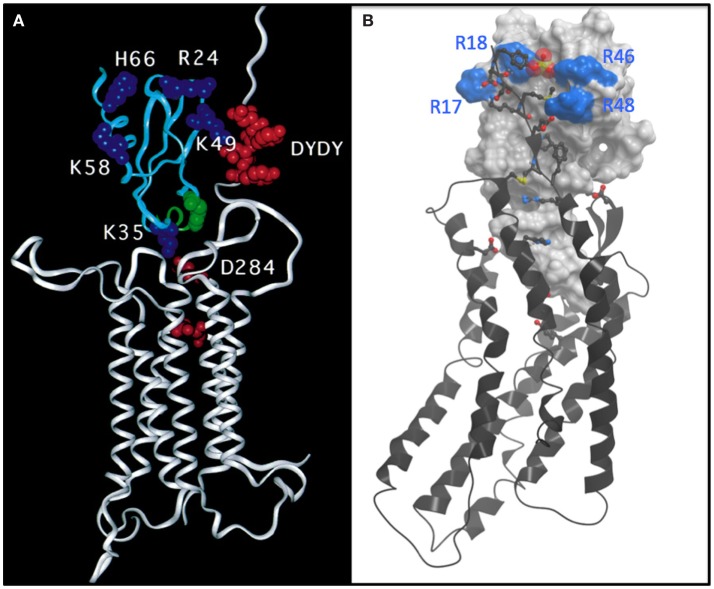
**(A)** Undocked model of the complex between CCR2 (white ribbon) and MCP-1 (cyan ribbon) based on mutagenesis and reproduced directly from Hemmerich et al. (12). The model features a number of basic residues (blue CPK) on MCP-1 that are important for binding. Acidic residues, particularly a DYDY tyrosine sulfation motif (red CPK) are highlighted on the N-terminus of CCR2. The model suggests how the DYDY motif might bind to a pocket on MCP-1 that is flanked by the basic residue cluster, similar to that shown **(B)**. **(B)** Structure of a CXCR4:vMIP-II complex where the N-terminus of CXCR4 is extended by two residues to include the sulfated tyrosine sTyr21 (13). vMIP-II is shown as a white mesh surface with basic residues colored blue. CXCR4 is shown as a black ribbon with acidic and basic side chains that make important interactions with vMIP-II shown as sticks with oxygens colored red and nitrogens colored blue. The sulfate group on Tyr21 is shown as a cluster of red and yellow spheres.

Because membrane receptors are so challenging, there was *no way* I was going to even consider working on intact chemokine receptor structures until/unless I got tenure at Berkeley, and fortunately that occurred in 2000. In 2002, I managed to hire a talented postdoc, Samantha Allen, from University of Bristol. She had a background in protein folding studies of bacteriorhodopsin, was interested in moving onto studies of eukaryotic membrane receptors and had the bravery (or perhaps naivety) to join me in the pursuit of chemokine receptor structures. Not having a track record in the expression, biochemistry or structural biology of membrane receptors, it was very difficult to get funding. Fortunately, Richard Horuk managed to convince his company, Berlex, to provide matching funds for a UC Discovery grant to pursue CCR1. It was not a lot of money, but that money along with fellowships that Samantha managed to garner, enabled us to hobble along. Samantha was ultimately able to express decent levels of CCR1 and to demonstrate reasonably high-affinity chemokine binding to purified receptor, and eventually we received a small NIH grant. However, CCR1 turned out to be a poor choice of receptor to pursue for structural studies. Chemokine receptors, like other GPCRs, are challenging, not only because they are membrane proteins but also because they are unstable and tend to fluctuate between multiple active and inactive conformations. As a consequence, they tend to aggregate when extracted from cell membranes unless heavily engineered and stabilized by ligands. CCR1 was on the wrong end of the challenge spectrum because it had an exceptionally high level of constitutive activity, which we discovered later, clued in by its poor biophysical behavior.

In 2005, I moved to University of California San Diego, to be with Peter Domaille, whom I married in 2004 (MCP-1 was definitely a chemoattractant!). Around 2008, I reconnected with Ray Stevens who had been at Berkeley when I started, but had moved to The Scripps Research Institute (TSRI). At TSRI, he had managed to build a rather large NIH-funded center, which later became the GPCR Network, with the goal of determining the structures of as many GPCRs as possible. We began working together and contributed to the first structure of CXCR4 with a cyclic peptide and small molecule antagonist, work that was spearheaded by his postdoc Beili Wu (14). This collaboration led to more substantial funding for my lab and our computational collaborators in the Abagyan group through an NIH funding mechanism called PSI:Biology. We were specifically paired with the GPCR Network as a “biological partner” to focus on determining structures of chemokine receptor complexes. People in my laboratory received training from the collective expertise of the GPCR Network team. We were then able to establish key elements of infrastructure (equipment, insect cell expression, biophysical assays) in our laboratory so that we could operate fairly independently, and we set our sights on determining the structure of CXCR4 with chemokine.

Compared to small molecule complexes with chemokine receptors or other GPCRs, which are challenging, complexes of CXCR4 with chemokine turned out to be even more difficult. The problem was that the detergent solubilized complexes were not sufficiently stable to survive crystallization conditions. We came to this conclusion after spending ~2 years using a strategy in which we made on the order of 100 mg of chemokine every 2 weeks to extract CXCR4 from membranes and to keep it stable during the purification process. This may make some people cry if they do the math, but recall that Peprotech was selling 50 μg of chemokine for ~$650 USD, and we were basically pouring it down the drain. Undeterred, but realizing that just adding chemokine to receptor was not the answer, we tried making fusions of chemokine to receptor; this strategy gave us sufficiently positive results to make us waste yet another year before giving up. Finally, I thought about the disulfide trap approach that Brian Kobilka had used to make a covalent complex of the β2-adrenergic receptor with a small molecule agonist (15); this seemed like an ideal approach for a receptor with a protein ligand because of the possibility of coexpressing single cysteine mutants of the receptor with cysteine mutants of the ligand. Moreover, because of my background in NMR, I thought it might provide a way of getting structural information in the form of disulfide-based distance restraints, even in the absence of a crystal structure. However, after all of these failures, imagine trying to convince your lab that the disulfide trap approach is a good idea, particularly when you do not know where to start! Fortunately, the lead post doc, Ling Qin accepted the challenge, although I am sure with considerable reluctance at first. Irina Kufareva, a computational chemist in the Abagyan lab was also on board and helped us identify an optimal disulfide pair through an iterative process of predicting potential disulfide pairs, experimentally testing coexpressed cysteine mutants of CXCR4 and chemokine for the presence and abundance of disulfide trapped complex, and evaluating the quality of the covalent complexes by various biophysical metrics. We pursued complexes of CXCR4 with both antagonist variants of the endogenous ligand CXCL12 (SDF-1) and the viral antagonist vMIP-II; antagonist ligands were chosen because we knew that WT CXCL12, an agonist, required G protein for high affinity, which would have added yet another enormous degree of complexity. Fortunately, in the first round of experiments with 11 different pairs, we identified one disulfide trap “hit” – just enough to be encouraging. Irina Kufareva was then able to use that hit as an experimental restraint in computational docking experiments to predict additional potential disulfide pairs, and eventually we identified a well-behaved complex of CXCR4 with vMIP-II, which crystallized (13) (Figure 1B). This structure explained a lot of biochemical data, and gave us insight into several other complexes including CXCR4 with CXCL12; it also provided insight into the specificity of CC versus CXC chemokines for their respective receptors, and further illustrated the structural plasticity of chemokine receptors, which enables them to recognize very different types of ligands. However, many more structures including agonist complexes will be required to fully understand how chemokines activate (or inhibit) their receptors, how even single amino acid changes can lead to changes in pharmacology (agonist versus antagonist responses), and the full basis of receptor:ligand specificity. Moreover, ternary complexes with intracellular signaling partners will be needed to understand the structural basis of the signaling and trafficking fate of receptors after chemokines bind, and how one can exploit this knowledge to develop drugs with finely tuned pharmacological properties.

The total elapsed time from the identification of the first disulfide trap to publication of the structure in January 2015 was ~2.5 years, but that was only after several years of failed strategies. Moreover, about 12 years elapsed between when we embarked on trying to express chemokine receptors for structural studies, and when we published the structure. During this time, I often wondered if I was out of my mind to go down this road; it certainly was not favorable for my publication record. I also wondered whether I should have taken over my grandmother’s ice cream business, “Handel’s,” instead of pursuing science. Hopefully, going forward, additional structures will yield to crystallization a little faster and with a little less sweat. Hopefully, the funding would not dry up before we complete at least a structure of MCP-1 with CCR2. And hopefully, these and other structures will aid in the development of drugs that target the chemokine receptor axis. Then it will all have been worth it.

## Conflict of Interest Statement

The author declares that the research was conducted in the absence of any commercial or financial relationships that could be construed as a potential conflict of interest.
